# DNA polymerases in biotechnology

**DOI:** 10.3389/fmicb.2014.00659

**Published:** 2014-12-01

**Authors:** Andrew F. Gardner, Zvi Kelman

**Affiliations:** ^1^New England Biolabs Inc.Ipswich, MA, USA; ^2^National Institute of Standards and TechnologyGaithersburg, MD, USA; ^3^Institute for Bioscience and Biotechnology ResearchRockville, MD, USA

**Keywords:** DNA polymerase, DNA polymerase evolution, DNA polymerase fidelity, DNA sequencing, molecular diagnostics, next generation sequencing, PCR, PCR inhibitors

Accurate duplication of parental DNA is a fundamental biological process, conserved in function across all life forms. All organisms depend on DNA polymerases for genome replication and maintenance. DNA polymerases also play central roles in modern molecular biology and biotechnology, enabling techniques including DNA cloning, the polymerase chain reaction (PCR), DNA sequencing, single nucleotide polymorphism (SNP) detection, whole genome amplification (WGA), synthetic biology, and molecular diagnostics. Each of these applications relies on the ability of polymerases to duplicate DNA, yielding a product that accurately represents the initial input. This book on “DNA Polymerases in Biotechnology” focuses on how detailed understanding of DNA polymerase structure and function informs protein engineering efforts, leading to development of novel reagents for molecular biology and clinical diagnostics.

DNA polymerases are classified into several families (A, B, C, D, X, Y) and reverse transcriptase (RT) based on primary amino acid sequence similarities (Burgers et al., 2001). The book leads off with several reviews that describe how these DNA polymerase families are evolutionarily (Makarova et al., 2014) and structurally (Doublie and Zahn, 2014) related as well as how polymerases have been utilized in biotechnology (Ishino and Ishino, 2014). Subsequent research articles build on this basic knowledge to describe how DNA polymerases are engineered as tools in biotechnology.

The best known and one of the earliest DNA polymerase-based biotechnology applications is PCR. Since its development over 30 years ago, PCR has been a foundational tool for amplifying and detecting specific alleles (Erlich et al., 1991). Advances in DNA polymerase fidelity, speed, and processivity continue to improve PCR workflows for genetic analysis, cloning, and diagnostics. Several articles in the issue highlight engineered polymerases with improved properties for PCR. Elshawadfy and co-workers demonstrate that combining desirable protein domains from several DNA polymerases into a single engineered chimeric enzyme can increase both speed and processivity during PCR (Elshawadfy et al., 2014). Similarly, Yamagami et al. create novel DNA polymerases by swapping domains from DNA polymerases found in hot springs to select for hybrid polymerases with desirable PCR properties (Yamagami et al., 2014). Castillo-Lizardo and colleagues analyze replication slippage during PCR of repeat sequences and show that the processivity clamp of DNA polymerase, proliferating cell nuclear antigen (PCNA) (Indiani and O'Donnell, 2006), reduces slippage to permit error-free replication of repeat sequences (Castillo-Lizardo et al., 2014).

As nucleic acid analysis by PCR moves toward clinical diagnostics, there is a need for both faster DNA polymerases and those that are capable of directly amplifying DNA from clinical samples such as tissue, blood, body fluids, or stool to speed and simplify diagnostic workflows. Several papers characterize DNA polymerases that tolerate PCR inhibitors and allow rapid DNA amplification from clinical samples without DNA purification, thereby reducing analysis time, cost, and potential for contamination. The contribution by Killelea et al. demonstrates that a Family D polymerase from *Pyrococcus abysii* is tolerant to high concentrations of PCR inhibitors while Arezi and colleagues describe a method to select for DNA polymerase variants that enable direct PCR from whole blood (Arezi et al., 2014; Killelea et al., 2014).

In addition to PCR, DNA polymerases play key roles in DNA sequencing technologies. Sanger DNA sequencing was used to sequence the first draft of the human genome in 2001 (Lander et al., 2001; Venter et al., 2001) and remains a standard and widespread method to determine DNA sequence. Several reviews describe the recent progress in the use of DNA polymerases for DNA sequencing. The review by Zhu examines the pivotal role of T7 DNA polymerase and its engineered derivatives in accelerating Sanger sequencing techniques (Zhu, 2014). Reha-Krantz et al. combine genetic and biochemical methods to identify T4 DNA polymerase mutants with increased processivity that along with T4 single stranded DNA binding protein (gp32) and T4 processivity factors (gp45 and gp44/62 complex) (Indiani and O'Donnell, 2006) improve Sanger sequencing of difficult DNA regions (Reha-Krantz et al., 2014). In the years since the human genome was first sequenced, new next generation sequencing methods have dramatically increased sequencing output while lowering costs (Mardis, 2011). Again, engineered DNA polymerases form the core of these next generation DNA sequencing-by-synthesis technologies. Chen reviews how DNA polymerases enable sequencing-by-synthesis technologies including the Illumina, Ion Torrent, and Pacific Biosciences platforms (Chen, 2014). The contribution by Laos et al. details how DNA polymerases have been engineered to incorporate the modified nucleotides used in DNA sequencing, genotyping, and synthesis of artificial DNA (Laos et al., 2014).

Recently, point of care diagnostic tests that are cheap, reliable and do not depend on specialized instruments have emerged. For example, isothermal amplification techniques such as Loop-Mediated Amplification (LAMP) have been routinely used as diagnostic tests to detect infectious disease (Njiru, 2012). Chander and co-worker describe an engineered thermostable viral polymerase with RT and DNA polymerase activities that can be used in isothermal RT-LAMP detection of RNA (Chander et al., 2014). Additionally, they demonstrate that the reaction components can be lyophilized as a dry pellet to allow storage without refrigeration and may be used in the field as a simple diagnostic test for RNA viruses.

## Future challenges

Engineered DNA polymerases will continue to play important roles in biotechnology and the delivery of health care. Over the next several years, molecular methods that are easier, cheaper, and faster will emerge. At the same time, molecular biology will move toward analysis of low concentration biomolecules (i.e., a single set of chromosomes). Unfortunately, tools for analysis of minute quantities of DNA are currently inadequate or technically challenging. For example, advances in sequencing technology (i.e., nanopore sequencing) can use extremely long DNA but methods to create long DNAs have not kept pace (Branton et al., 2008; Metzker, 2010; Shendure et al., 2011). Novel amplification techniques are also required to profile genetic variations among single cells (Navin and Hicks, 2011; Schubert, 2011) because the quantity of genomic DNA from a single cell is insufficient to sequence directly. Therefore, DNA must first be amplified prior to further analysis (Kalisky and Quake, 2011; Kalisky et al., 2011).

Additionally, synthetic biology aims to design new biological systems such as genetic pathways, operons, and genomes (Montague et al., 2012) and thus may require long, chromosome-size, amplification. Pathway engineering relies on assembling the DNA coding for the desired characteristics and then using a host to activate the pathway *in vivo*. Current methods for DNA assembly are limited to about 20 kb and larger fragments must be assembled *in vivo* at a very low frequency thereby limiting utility. Furthermore, current DNA polymerases introduce errors during amplification and thus DNA polymerases with very low error rates are needed to ensure that long, amplified DNA are exact copies of the starting material.

Therefore, novel DNA amplification systems are needed to accelerate progress in emerging technologies and to make high-fidelity *in vitro* genome analysis and manipulation routine. Engineered DNA polymerases or cellular replication machineries capable of amplifying large DNA fragments have the potential to enable single cell genomics, genome synthesis, and manipulation. This issue summarizes the known properties of various DNA polymerase systems and how DNA polymerases are currently being manipulated to meet these growing demands.

### Conflict of interest statement

The authors declare that the research was conducted in the absence of any commercial or financial relationships that could be construed as a potential conflict of interest.
